# Chondroitinase ABC in spinal cord injury: advances in delivery strategies and therapeutic synergies

**DOI:** 10.3389/fbioe.2025.1604502

**Published:** 2025-06-09

**Authors:** Rachel Santana Cunha, Erik Aranha Rossi, Thaís Alves de Santana, Zaquer Suzana Munhoz Costa-Ferro, Bruno Solano de Freitas Souza

**Affiliations:** ^1^ Center for Biotechnology and Cell Therapy, São Rafael Hospital, Salvador, Bahia, Brazil; ^2^ CBTC, D´Or Institute for Research and Education (IDOR), Salvador, Brazil; ^3^ Gonçalo Moniz Institute, Oswaldo Cruz Foundation (FIOCRUZ), Rio de Janeiro, Bahia, Brazil; ^4^ Pioneer Science Initiative, D’Or Institute for Research and Education (IDOR), Rio de Janeiro, Brazil

**Keywords:** spinal cord injury, chondroitinase ABC, neural regeneration, delivery, gene therapy, nanotechnology, stem cell therapy

## Abstract

Spinal cord injury (SCI) is a debilitating condition that leads to permanent neurological deficits due to the formation of a glial scar and the accumulation of chondroitin sulfate proteoglycans (CSPGs), which inhibit axonal regeneration. Chondroitinase ABC (ChABC), a bacterial enzyme capable of degrading CSPGs, has emerged as a promising therapeutic strategy for enhancing neural plasticity and functional recovery after SCI. However, clinical translation remains challenging due to the enzyme’s thermal instability, short half-life, and limited penetration into the lesion site. This review provides a comprehensive overview of current strategies for ChABC delivery, including direct infusion, nanoparticles, hydrogels, scaffolds, viral vectors, and stem cell-based approaches. We highlight recent technological advances that improve enzyme stability, targeting, and sustained release, as well as combinatorial therapies that enhance tissue regeneration. Although ChABC monotherapy has shown limited efficacy, its association with other regenerative approaches has demonstrated significant potential in preclinical models. Finally, we discuss the translational challenges and future directions required to bring ChABC-based therapies closer to clinical application in SCI patients.

## 1 Introduction

Spinal cord injury (SCI) is a neurological condition caused by damage to the spinal cord, resulting in temporary or permanent functional impairments. It has devastating and long-term physical, psychological, and socioeconomic consequences for patients and their families. SCI can result from high-intensity mechanical trauma, such as traffic accidents, or from infections, tumors, and degenerative disorders. These injuries often lead to severe disability, including motor and sensory deficits, urinary and sexual dysfunction, respiratory impairment, and chronic neuropathic pain (Ahuja et al., 2017). Globally, SCI affects more than 15 million people, with a higher incidence among working-age individuals (WHO, 2024). This prevalence places a substantial financial burden on healthcare systems, as current treatments are limited and primarily offer supportive care for lifelong disabilities. It is estimated that the average lifetime cost per patient can reach up to 2.5 million dollars (Diop and Epstein, 2024).

Regarding functional recovery, many studies have identified a common challenge: although central nervous system (CNS) neurons attempt to regenerate after traumatic injury, the post-injury environment is highly inhibitory, leading to abortive regeneration (Bradbury and Burnside, 2019). This is primarily due to the complex pathophysiology of the CNS, which undergoes significant biochemical and structural alterations following injury. The initial trauma causes extensive tissue damage, disrupts the blood-brain/spinal cord barrier, and leads to necrotic cell death. The compromised vasculature facilitates an influx of inflammatory cells that release pro-inflammatory cytokines and vasoactive peptides, which exacerbate damage through mechanisms such as edema, excitotoxicity, altered gene expression, and dysregulated cellular signaling. Moreover, reactive astrocytes surround the lesion site and secrete a variety of pro-inflammatory molecules, as well as extracellular matrix components such as chondroitin sulfate proteoglycans (CSPGs), leading to the formation of a dense glial scar. This scar acts as both a mechanical and chemical barrier to axonal regeneration (Sofroniew and Vinters, 2010).

Chondroitinase ABC (ChABC) is a bacterial enzyme derived from *Proteus vulgaris* that degrades the sulfated chondroitin chains of CSPGs. Due to its ability to modify the inhibitory extracellular matrix, ChABC has been extensively investigated in experimental therapies for CNS injuries, especially SCI. However, under physiological conditions, ChABC is rapidly degraded, limiting its therapeutic potential. For clinical applications, local and sustained delivery is necessary to maintain its activity (Letko Khait et al., 2025). Over the years, researchers have increasingly explored the combination of ChABC with other strategies, such as cell therapy, which has shown to enhance tissue repair and functional recovery. Current research efforts are now focused on improving the stability of the enzyme and developing effective delivery systems. Therefore, this review aims to explore the different delivery strategies for ChABC, draw comparisons between these approaches, and discuss the implications of combining ChABC with other therapies for the treatment of spinal cord injury.

## 2 Pathophysiology of SCI

Under normal conditions, the spinal cord is a complex environment characterized by intricate molecular pathways and dynamic interactions among various cell types, including astrocytes, neurons, microglia, and oligodendrocytes (Anjum et al., 2020). When an injury occurs, these interactions are disrupted or disorganized, impairing communication between the brain and the rest of the body. This disruption leads to loss of sensation, movement, and reflexes below the level of the injury (Lu et al., 2023).

The initial mechanical trauma to the spinal cord is referred to as the primary injury. It may be caused by contusion, laceration, or compression, or may result from diseases such as cancer or various syndromes (Alizadeh et al., 2019). This primary insult results in destruction of the neural parenchyma and initiates a cascade of secondary events, including axonal damage, hemorrhage, blood-spinal cord barrier (BSCB) disruption, ionic imbalance, glutamate excitotoxicity, infiltration of inflammatory cells, and the activation of resident glial cells. Consequently, a series of biochemical, mechanical, and physiological changes ensue, further compromising spinal cord function. This cascade is known as secondary injury (Anjum et al., 2020).

The secondary injury is considered the major contributor to tissue loss and neurological dysfunction after SCI. It is characterized by an intense inflammatory response that increases cell permeability, apoptosis, ischemia, vascular damage, edema, oxidative stress, and the release of inflammatory cytokines. These effects not only damage cells at the injury site but also affect neighboring regions (Anjum et al., 2020). These events span the acute and subacute phases of SCI and taper off during the chronic phase, during which a glial scar forms—one of the major impediments to axonal regeneration (Raspa et al., 2021).

The glial scar forms in response to cellular signals released around the injury epicenter. These signals activate glial cells, particularly astrocytes, triggering a process known as reactive gliosis (Clifford et al., 2023). To stabilize the injury site, astrocytes and fibroblasts produce extracellular matrix components, including CSPGs (Galtrey and Fawcett, 2007). While this process serves a protective role by limiting further tissue damage, it also creates a dense barrier that hinders axonal reconnection and cell migration. Thus, the glial scar becomes a major contributor to motor function loss in patients with SCI (Zweckberger et al., 2016).

Interestingly, studies have shown that CSPGs produced by astrocytes and fibroblasts can be degraded by ChABC, a bacterial enzyme capable of cleaving glycosaminoglycan side chains on proteoglycans (Raspa et al., 2021). This unique property has sparked significant interest in using ChABC to promote neural regeneration in SCI, as its ability to remove inhibitory substrates allows the formation of new neural connections and holds therapeutic potential for functional recovery.

## 3 Challenges in ChABC delivery

A major hurdle in translating ChABC therapy to the clinic is achieving efficient and long-lasting delivery of the enzyme at the SCI site, due to its instability and short half-life *in vivo*. Systemic delivery is hindered by the BSCB, which restricts the passage of large or hydrophilic molecules, such as enzymes, necessitating local or vector-mediated strategies. Although most current strategies rely on local delivery to bypass this barrier, challenges remain even with direct intrathecal or intraparenchymal administration. These include limited enzyme diffusion within the dense, fibrotic, and irregular structure of the glial scar, as well as difficulty in achieving uniform distribution throughout the lesion area, short half-life of the enzyme, and the need for repeated invasive administrations (Jin et al., 2021).

Once injected, ChABC can be rapidly dispersed or cleared, and its enzymatic action may be confined to the immediate vicinity of the injection site, leaving other areas of the lesion inadequately treated. In addition, the complexity of the post-injury microenvironment—including extracellular matrix (ECM) compaction, altered pH, and inflammatory activity—can further restrict enzyme penetration and activity. Intrathecal injection, although widely studied, may result in suboptimal enzyme distribution due to dilution and cerebrospinal fluid dynamics, requiring either high doses or repeated applications. However, high concentrations (e.g., >50 U) have been associated with complications such as subarachnoid hemorrhage in some animal models (Cheng et al., 2015). Moreover, repeated injections increase the risk of tissue damage, inflammation, and infection (Lee et al., 2010). Another limiting factor is the rapid inactivation of ChABC at physiological temperature and pH. The enzyme loses significant activity within hours at 37°C, while the pathological environment after SCI—characterized by CSPG accumulation and scar formation—persists for weeks (Kosuri et al., 2022).

Therefore, a single administration is rarely sufficient to achieve meaningful tissue remodeling and functional recovery. These obstacles have prompted the development of novel delivery strategies aimed at improving enzyme stability, retention, targeting, and tissue penetration. Approaches include the encapsulation of ChABC in nanoparticles and hydrogels for sustained release, as well as gene therapy using viral vectors to provide continuous local expression of the enzyme (Jin et al., 2021; Kosuri et al., 2022; Hettiaratchi et al., 2020). Each strategy seeks to address key pharmacokinetic and biodistribution limitations of ChABC, paving the way toward its clinical translation.

## 4 Methods for ChABC delivery

**FIGURE 1:**
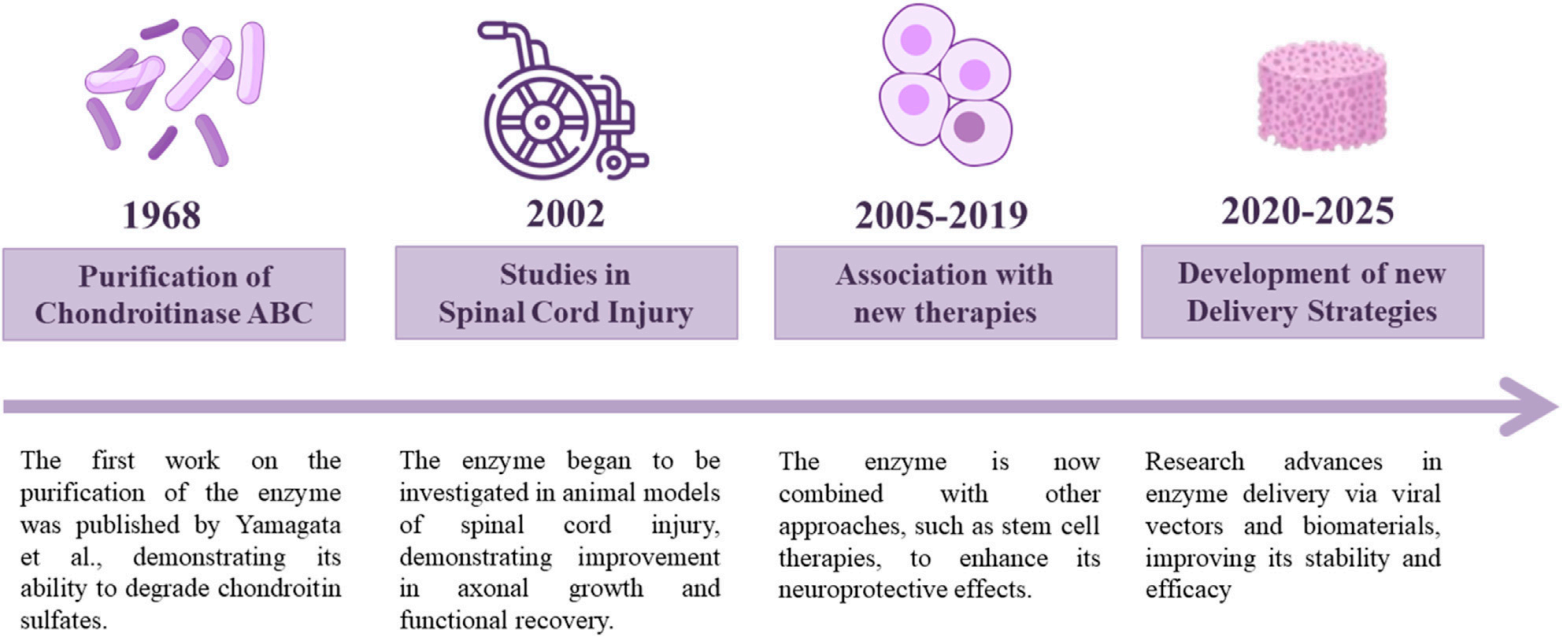
ChABC discovery and therapeutic development timeline. Since the discovery of ChABC in 1968, many studies have been performed focusing on enhancing its potential for SCI treatment.

**FIGURE 2:**
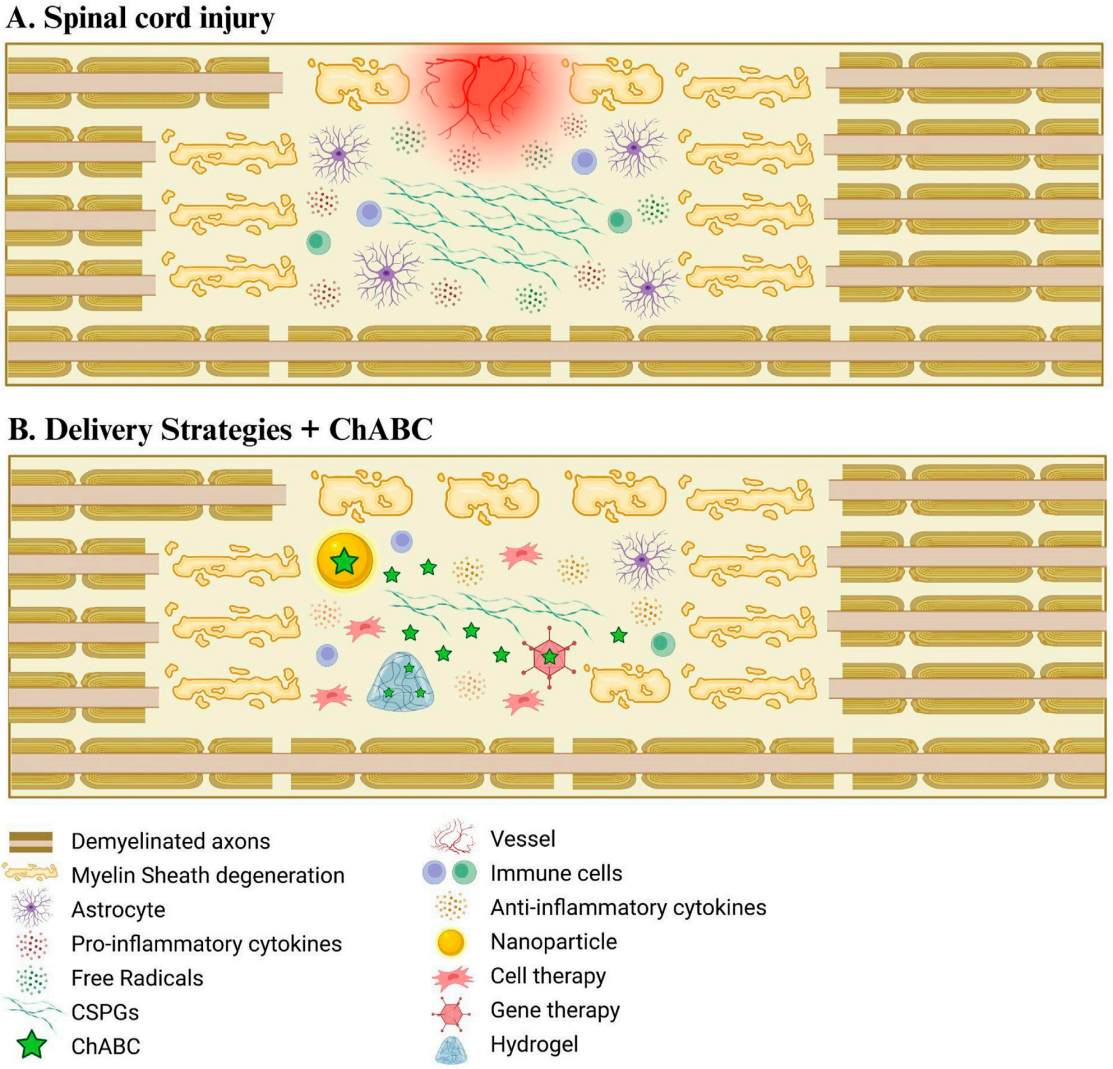
Delivery strategies of ChABC for spinal cord injury treatment. **(A)** Schematic drawing representing spinal cord injury damage. **(B)** The combination of different delivery systems with ChABC enhances the recovery of the injured environment, enabling a more efficient degradation of CSPGs.

Following the discovery of ChABC by Yamagata et al. (1968), who demonstrated its ability to degrade chondroitin sulfates (Yamagata et al., 1968), numerous studies have explored its therapeutic potential. In 2002, investigations into its effects on SCI began to show promising outcomes, prompting further research into combination therapies aimed at enhancing its regenerative effects (Figures 1, 2).

ChABC treatment has been investigated in both the acute and chronic phases of SCI, showing potential to offer significant therapeutic benefits in either context. In the acute phase, ChABC may exert its effects by modulating the early inflammatory environment and limiting the formation of reactive gliosis (Anjum et al., 2020). The immunomodulatory properties of ChABC were investigated by Didangelos and colleagues (2014), who reported an increase in IL-10 expression and reduction in the pro-inflammatory cytokine IL-12B following enzyme administration. The same study also reported increased infiltration of M2-polarized macrophages into the injured tissue, although the underlying mechanisms remain poorly understood. Similarly, Akbari et al. (2017) demonstrated that ChABC mitigates inflammation and oxidative stress associated with SCI, with reductions in TNFα, IL1β, and nitric oxide levels.

Despite these promising findings, the majority of SCI patients are in the chronic phase, reinforcing the importance of long-term preclinical studies to evaluate the *in vivo* efficacy of ChABC. In this phase, the presence of a mature glial scar represents a major barrier to tissue regeneration, forming a dense and inhibitory environment that necessitates strategies capable of sustaining enzymatic activity over extended periods. Therefore, sustained delivery systems for ChABC - such as hydrogels or viral vectors - have been associated with long-lasting modifications of the spinal microenvironment. Several studies have reported persistent reductions in glial fibrillary acidic protein (GFAP) and CSPG expression up to several weeks post-treatment (Milbreta et al., 2014; Raspa et al., 2018; Hlavac et al., 2021), indicating continued suppression of astrogliosis. This remodeling not only facilitates axonal sprouting but, in cell therapy studies, also promotes greater integration of transplanted cells, laying the groundwork for synergistic effects in combination therapies. Transplanted cells can have their effect enhanced by pre-treatment with ChABC. By degrading CSPGs and attenuating the glial response, ChABC creates a more permissive environment for cell engraftment. This enzymatic action re-exposes key extracellular matrix components, such as laminin and fibronectin, which support cell adhesion and neurite outgrowth (Suzuki et al., 2017). In addition to reducing biochemical inhibition, CSPG degradation physically clears space for tissue repopulation.

Regarding combined therapies, several studies suggest that ChABC acts synergistically rather than merely additively. For instance, Suzuki et al. (2017) reported ChABC pre-treatment in a severe chronic SCI model increased the survival rate of grafted iPSC-derived neural stem cells (iPSC-NSCs) from 2.44% ± 1.04% to 7.88% ± 1.60%, representing more than a threefold improvement compared to transplantation alone. Lu and collaborators (2020) observed an approximately 70% increase in adipose-derived mesenchymal stem cell migration following enzymatic pre-treatment, further supporting a synergistic mechanism. These findings emphasize the importance of tailoring ChABC-based therapies to the temporal stage of the injury and leveraging synergistic mechanisms for optimal functional recovery. Table 1 presents an overview of preclinical studies using ChABC for SCI. It includes the injury model, timing of intervention, route of administration, and the type of ChABC formulation/delivery strategy used.

**TABLE 1:** Summary of experimental models of Spinal Cord Injury.

Animal model	Injury model	Phase	Route of administration	ChABC delivery strategy	Study
Sprague Dawley rats	Contusion at T10/11	Acute	Intraspinal injection	*In vivo* delivery of a lentiviral vector carrying ChABC gene	Bartus et al. (2014)
Sprague Dawley rats	Contusion at C5	Acute	Intraspinal injection	*In vivo* delivery of a lentiviral vector carrying ChABC gene	James et al. (2015)
Sprague Dawley rats	Contusion at T10	Acute	Intraspinal injection	Transplantation of ADSCs genetically modified *ex vivo* using a lentiviral vector to express ChABC	Lu et al. (2020)
Sprague Dawley rats	Contusion at T9/10	Chronic	Intraspinal injection	Self-assembling peptide hydrogels carrying recombinant ChABC	Raspa et al. (2021)
Sprague Dawley rats	Contusion at T10	Acute	Intralesional injection	Nonviral delivery of ChABC mRNA using mineral-coated microparticles	Khalil et al. (2022)
Wistar rats	Contusion at T10	Acute	Intraspinal injection	PLGA nanoparticles with ChABC	Azizi et al. (2020)
Wistar rats	Compression at C3	Acute	Midline injections	mOECs expressing ChABC	Prager et al. (2021)
Wistar rats	Compression at T13/L1	Acute	Intraspinal injury	ChABC combined with low-level laser therapy	Janzadeh et al. (2017)
Lister Hooded rats	Contusion at C5/6	Acute-Chronic	Intraspinal injection	Dual lentiviral immune-evasive doxycycline-inducible ChABC (dox-i-ChABC) vector system	Burnside et al. (2018)
Dogs	Compression at L4	Chronic	Intraspinal injection	Recombinant ChABC + transplantation of MSCs	Lee et al. (2015)
Dogs	Complete motor and sensory loss a minimum of 3 months post-injury - different injury levels	Chronic	Intrathecal injection	Encapsulated mOECs expressing ChABC	Prager et al. (2022)
Rhesus monkeys	Lateral hemisection at C7	Acute	Intraparenchymal injection	Recombinant ChABC	Rosenzweig et al. (2019)
Lampreys	Transection injury	Acute - Chronic	Intralesional Gelfoam pledget	Antisense oligonucleotides (RhoA knockdown) + ChABC	Hu et al. (2023)

### 4.1 Direct infusion of recombinant ChABC

Initial studies with ChABC focused on its direct administration to the injury site, either intraparenchymal or intrathecally. This method allows bypassing systemic barriers such as the BSCB and delivers the enzyme directly to the region of interest. To enhance the therapeutic effects of the direct infusion of ChABC, some studies have explored its combination with other agents that promote regeneration. For instance, Yick and colleagues (2004) compared the effects of ChABC alone and in combination with lithium chloride. Lithium’s beneficial effect in this context is attributed to its inhibition of glycogen synthase kinase-3β (GSK-3β), which enhances axonal growth and neuronal survival through activation of pro-regenerative signaling pathways, such as Wnt/β-catenin. The authors found that ChABC alone promoted a 20% increase in axonal regeneration in the rubrospinal tract, while the combined therapy yielded a 42% increase, highlighting both the independent and synergistic effects of the enzyme (Yick et al., 2004).

The effectiveness of ChABC delivery can also be influenced by anatomical variables related to the site of injection. Tom et al. (2009) investigated the outcomes of ChABC injection rostrally *versus* caudally to the lesion site. Results showed that rostral injection led to a significant increase in 5HT + fibers in the dorsal and ventral horns, whereas caudal injection did not produce similar outcomes. These findings emphasize how anatomical barriers and cerebrospinal fluid flow dynamics can influence enzyme distribution, highlighting the importance of precise targeting and optimized delivery strategies (Tom et al., 2009).

Despite the advantages of local delivery, direct infusion of ChABC faces several limitations. The enzyme is unstable at physiological temperature, with its activity decreasing significantly within hours of administration. Moreover, its diffusion within the lesion site is often restricted due to the dense extracellular matrix and compact glial scar, resulting in heterogeneous tissue exposure and incomplete CSPG degradation. As a result, multiple or continuous infusions would be required to maintain enzymatic activity over the time course of glial scar formation, which typically extends over several weeks (Tester and Howland, 2008; Lin et al., 2008).

Cheng et al. (2015) investigated ChABC delivery at different time points and concentrations. Intrathecal administration of 50 U during the acute phase of SCI led to adverse outcomes, including subarachnoid hemorrhage and mortality within 48 h. In contrast, during the subacute phase, lower (1–5 U) and higher (50–100 U) doses were tested with safer profiles, and only the higher doses induced significant functional recovery (Cheng et al., 2015). This study illustrates the delicate balance between therapeutic efficacy and safety, influenced by both dose and timing of administration. An alternative strategy involves the use of programmable microinfusion pumps to ensure continuous and localized enzyme delivery over days or weeks, but such devices have limited translational relevance, as their complexity and risk profile are incompatible with clinical application in the CNS. Furthermore, the need for frequent invasive procedures poses a significant barrier to clinical translation, particularly in patients with chronic SCI (Lee et al., 2010).

Although direct infusion has shown significant functional improvements in preclinical models - including enhanced axonal regeneration, synaptic reorganization, and partial recovery of locomotor and sensory function (Barritt et al., 2006; Carter et al., 2008; Mondello et al., 2015) - its limitations in terms of stability, spatial distribution, and clinical feasibility have prompted the search for improved delivery platforms. As such, strategies like encapsulation, hydrogel-mediated release, and gene therapy have gained increasing attention in the field.

Targeted drug delivery involves directing the therapeutic agent to specific cells, tissues, or organs using receptor-ligand interactions, often through carriers that accumulate and release the drug at the desired site. This approach aims to eliminate or reduce the accumulation of a medication in tissues that do not add to the therapy, thereby increasing bioavailability and reducing side effects (Lu et al., 2023). Nanodelivery systems, in particular, offer targeted and controlled release and represent a promising strategy for ChABC delivery (Chen, 2022).

The use of a vehicle can result in greater potency of the therapeutic effects and reduction of adverse effects as demonstrated by Wiklander et al. (2024), where a cytotoxic drug was combined with an engineered nanostructure, an extracellular vesicle. From the elaborate construction, there was a targeting of the drug to the affected region and greater potential of the drug, which means that lower concentrations can be used to obtain the desired effect and less risk to the patient (Wiklander et al., 2024). Moreover, this association may represent a future strategy for the targeted delivery of ChABC.

### 4.2 Controlled release systems: nanoparticles and hydrogels

Advances in nanotechnology associated with molecular biology have enabled the application of nanoscale biomaterials that contribute as a vehicle for the transport and controlled delivery of drugs (Chen, 2022). Controlled release systems have emerged as promising strategies to overcome the limitations associated with direct ChABC infusion, particularly those related to enzyme instability, rapid degradation, and uneven tissue distribution. By encapsulating the enzyme within biocompatible carriers, such as nanoparticles or hydrogels, these systems aim to preserve its bioactivity, extend its therapeutic window, and improve tissue distribution while minimizing invasive procedures. Among the most studied delivery platforms are biodegradable nanoparticles and hydrogels, which can encapsulate ChABC and release it over extended periods.

Nanoparticles, particularly those made from biodegradable polymers such as poly (lactic-co-glycolic acid) (PLGA), offer several advantages including controlled degradation, surface modification capabilities, and protection of labile biomolecules (Costăchescu et al., 2022). Azizi et al. (2020) formulated PLGA nanoparticles containing ChABC using water-in-oil-in-water (W/O/W) emulsions, protecting the enzyme from degradation and enabling controlled release. Their study showed that animals receiving the nanoparticle formulation exhibited greater glial scar degradation and improvements on the Basso, Beattie, and Bresnahan (BBB) scale during the first 5 weeks of treatment, indicating recovery of locomotor function (Azizi et al., 2020).

Hydrogels are three-dimensional networks formed by cross-linked polymers capable of absorbing large amounts of fluid (Gao et al., 2016). Among them, self-assembling peptide hydrogels (SAPs) have emerged as a promising option for sustained ChABC release. Raspa et al. (2021) evaluated two different SAP-based nanostructures for ChABC delivery: in one approach, the enzyme was mixed with SAPs prior to gelation; in the other, ChABC was injected into a pre-formed gel. While typical *in vitro* activity of ChABC lasts around 72 h, combining the enzyme with these hydrogels enabled continuous release for up to 42 days, demonstrating a significant improvement in enzyme stability and sustained bioavailability.

In addition to hydrogels, scaffolds can also enhance ChABC stability and allow for sustained release into injured tissue. This continuous delivery helps maintain high levels of bioactive ChABC at the lesion site over extended periods (Sharifi et al., 2022; Raspa et al., 2018). One advantage of scaffolds is the ability to manipulate their morphology and composition to optimize the microenvironment for enzymatic activity. Scaffolds can be combined with nanoparticles, which have been shown to reduce oxidative stress following SCI (Chen et al., 2021) and to promote axonal outgrowth (Zuidema et al., 2016). Furthermore, both natural biomaterials and synthetic materials can be used in scaffold design. While both are biodegradable and support neural cell attachment in SCI models involving hemisection or transection, synthetic materials offer the advantage of greater availability and tunable degradation rates (Chen et al., 2021). These properties make scaffolds a promising approach for clinical delivery of ChABC, as they address the enzyme’s instability while contributing to tissue regeneration, depending on the scaffold’s composition.

Hlavac et al. (2021) evaluated a novel strategy combining enzyme engineering with scaffold-based delivery. In their study, ChABC was fused to galectin-3 (Gal3) and applied to hyaluronan (HA)-based scaffolds. Gal3 binds β-galactoside glycans present on cell surfaces and extracellular matrix components, such as CSPGs, and its fusion to ChABC was designed to enhance tissue retention. HA was selected for its neurocompatibility and ability to serve as both a release vehicle and a temporary scaffold for regenerating cells following scar degradation. To enhance bioactivity, the scaffolds were supplemented with collagen I and laminin, promoting cell adhesion and differentiation. ChABC-Gal3 released from this system maintained enzymatic activity and CSPG degradation capacity for at least 10 days—significantly longer than wild-type ChABC, which typically loses function after 1 day. These results suggest that this strategy is more effective in reducing scar tissue and improving local enzyme retention (Hlavc et al., 2021).

Although *in vitro* and animal studies have yielded promising results, further research is needed to assess the translatability of these systems in large animal models and clinical settings. Prager et al. (2022) investigated the feasibility of intraspinal transplantation of autologous mucosal olfactory ensheathing cell (mOEC) populations expressing ChABC and encapsulated in collagen hydrogel in dogs with naturally occurring spinal cord injuries. Following olfactory mucosa biopsy, mOECs were cultured, genetically modified to express ChABC, and delivered percutaneously via hydrogel injection into the spinal cord. Post-transplantation magnetic resonance imaging showed no signs of compression or ischemia. While gait and kinematic analyses showed no statistically significant improvement, dog owners reported enhanced pelvic limb reflexes, and some animals regained the ability to take 2–3 steps without support. These findings highlight the clinical potential of combining gene-modified cells with biomaterial-based delivery platforms for ChABC therapy (Prager et al., 2022).

### 4.3 Genetic engineering and viral vectors

The use of viral vectors, such as lentivirus (LV) and adeno-associated virus (AAV) systems, has emerged as a more efficient strategy for delivering ChABC, particularly in terms of achieving sustained expression at the lesion site. Experimental studies have demonstrated that these systems can preserve ChABC activity *in situ* for periods exceeding 4 weeks (Zhao and Fawcett, 2013). Viral vectors support long-term ChABC release, as transduced cells can continuously synthesize the enzyme, reducing the need for multiple injections. This is particularly advantageous when considering the clinical translation of this approach (Islam and Tom, 2022).

Both lentiviral (LV) and adeno-associated virus (AAV) vectors can successfully transfer genes into dividing and non-dividing cells. AAV vectors have a favorable safety profile for *in vivo* applications, with minimal systemic immune activation, while the integrative nature of LVs raises concerns about insertional mutagenesis, particularly for direct *in vivo* use (Gao et al., 2002; Zheng et al., 2018). This limitation was addressed in a study employing an immune-evasive dual-vector LV system incorporating a chimeric transactivator designed to evade T cell recognition, which resulted in improved sensory axon conduction and motor function in a rat model of cervical contusion injury (Burnside et al., 2018). While LVs offer the advantage of stable, long-term gene expression—including in non-dividing cells—their immunogenicity may vary depending on the pseudotype and target tissue (Abordo-Adesida et al., 2005). In contrast, AAVs are non-integrative, substantially reducing the risk of insertional mutagenesis, and are generally associated with lower immunogenicity *in vivo* (Wu et al., 2024a).

Building upon the favorable properties of these vectors, several studies have employed gene therapy approaches to modulate secondary injury mechanisms and promote functional recovery after spinal cord injury. For instance, Bartus et al. (2014) and James et al. (2015) demonstrated improvements in sensorimotor function and increased serotonergic innervation following gene-based interventions (Bartus et al., 2014; James et al., 2015). To further enhance the specificity of gene delivery, Carstens et al. (2021) designed an AAV vector encoding ChABC under the control of the Cre-LoxP system. This approach enabled ChABC expression specifically in neurons that expressed the Cre protein *in vivo*. As a result, targeted delivery was achieved, and when various regions of the hippocampus were compared, the CA2 region—selected as the target—exhibited a marked reduction in perineuronal nets, confirming ChABC activity (Carstens et al., 2021).

Despite these advantages, the use of viral vectors for ChABC delivery still presents certain drawbacks when compared to other delivery systems such as scaffolds, hydrogels, and nanomaterials. These include a residual risk of oncogenicity, as well as the technical complexity associated with vector production and application (Parr-Brownlie et al., 2015). Therefore, while viral vectors offer strong potential, alternative strategies may be more suitable for clinical use, depending on the context and therapeutic goals.

### 4.4 Delivery by stem cells

Among the different strategies aimed at restoring the injured spinal cord environment, one involves the *ex vivo* overexpression of ChABC in different cell types, including stem cells, followed by cell transplantation and engraftment. Alternatively, unmodified cells can be associated with ChABC treatment using different methodologies. The excessive accumulation of CSPGs following injury negatively affects the survival and migration of transplanted cells. Therefore, combining cell therapy with ChABC presents a promising therapeutic strategy (Lu et al., 2020). For transplanted cells to function effectively, a favorable environment that supports migration is essential, which requires the degradation of CSPGs.

Among the various cell types, neural stem cells (NSCs) are particularly relevant due to their ability to differentiate into all three neuroglial lineages, contribute to neural circuit regeneration, remyelinate axons, and provide trophic support for endogenous cells (Suzuki et al., 2017). In a study conducted by Suzuki et al. (2017), ChABC pretreatment was administered first, followed 1 week later by transplantation of NSCs derived from induced pluripotent stem cells (iPSCs). As expected, the pretreatment significantly enhanced the survival of iPSC-NSCs at 8 weeks post-transplantation, particularly promoting the proliferation of acetylcholinergic neurons, which are critical for motor function. In another approach, Nori et al. (2018) utilized oligodendrogenic neural progenitor cells (oNPCs) in combination with sustained ChABC delivery via a methylcellulose biomaterial. This combinatorial therapy led to increased long-term survival of oNPCs near the injury epicenter, enhanced oligodendrocyte differentiation, remyelination of spared axons by the grafted cells, increased synaptic connectivity with anterior horn cells, and improved neurobehavioral outcomes (Nori et al., 2018). Thus, this combined therapy appears to be a promising strategy for regenerating the chronically injured spinal cord.

Schwann cells (SCs) are also considered valuable agents for restoring the injured environment, particularly by promoting remyelination. Once the inhibitory environment is alleviated, promoting remyelination becomes essential. Qu and colleagues (2025) evaluated the effects of SC transplantation into the lesion epicenter, combined with lentivirus-mediated ChABC delivery rostral and caudal to the injury site. This combined strategy promoted glial scar degradation, Schwann cell migration, axonal regeneration, and recovery of both locomotor and urinary bladder function. Importantly, the study also addressed treatment in the chronic phase: 3 months post-injury, the combined SC + lenti-ChABC therapy was administered and monitored for up to 6 months. The results demonstrated SC survival and sustained axonal growth throughout the follow-up period, representing a significant therapeutic achievement (Qu et al., 2025).

Mesenchymal stem/stromal cells (MSCs) have gained prominence in spinal cord injury research over the past decades, and their use in clinical trials is already a reality (Bydon et al., 2024; Macêdo et al., 2024). Moreover, the combination of MSCs with ChABC has been evaluated in preclinical models. Lu and colleagues (2020) modified adipose-derived mesenchymal stem cells (ADSCs) to overexpress ChABC. Four weeks after transplantation, the CSPG content was significantly reduced in the group that received the modified ADSCs, demonstrating enhanced proteoglycan degradation. Furthermore, improved motor function recovery was observed in the hind limbs of the treated animals. In addition to promoting CSPG degradation, this strategy also resulted in approximately 70% increased migration of the transplanted cells to the injury site (Lu et al., 2020). In a complementary approach, Lee et al. (2015) used a canine SCI model and treated the animals with adipose tissue-derived MSCs combined with ChABC. Eight weeks post-transplantation, the group receiving the combined therapy exhibited more pronounced functional recovery and clinical improvement, in addition to increased CSPG digestion (Lee et al., 2015). In another study, Prager and colleagues (2021) explored the synergy between ChABC and canine mucosal olfactory lining cells, which were genetically modified via lentiviral transduction to express ChABC. This system enabled sustained enzyme release and significantly improved motor recovery, yielding greater efficacy than treatments using either component alone (Prager et al., 2021).

Table 2 provides a comprehensive overview of ChABC-based studies conducted over the years, summarizing various delivery methods employed for SCI treatment. The data highlight improvements in motor function, axonal regeneration, and overall functional recovery, while also addressing challenges such as outcome variability, enzyme stability, and the necessity for complementary therapeutic approaches. This compilation serves as a valuable reference for understanding both the advancements and the ongoing limitations of ChABC-based therapies in SCI research. Following this analysis of the most used methods for ChABC delivery, Table 3 summarizes the main advantages and disadvantages of each strategy reviewed in this article.

**TABLE 2:** ChABC delivery strategies by study: main outcomes and limitations.

Delivery strategy	Model	Main outcomes	Limitations	Study
Micro/Nanoparticles	Rat - T10 contusion	Improved motor recovery; reduced astrocytic response and CSPG expression	PLGA-related acidification may affect stability (not evaluated)	Azizi et al. (2020)
	*In vitro* Schwann cells	Increased Schwann cell migration	*In vitro* only; lacks validation in animal models	Gao et al. (2020)
	Rat - T10 contusion	Enhanced motor function; decreased inflammation; efficient mRNA delivery	Long-term stability remains uncertain; Transient expression from mRNA	Khalil et al. (2022)
Hydrogels	Rat - T9/10 contusion	Sustained ChABC release (42 days); neural regeneration	Thermal stability needs improvement; diffusion from application site	Raspa et al. (2021)
	*In vitro* Dorsal ganglia neurons	Effective CSPG degradation; axonal growth promotion	No *in vivo* validation	Hlavac et al. (2021)
	Dog - chronic SCI (varied levels)	Safe and feasible administration, mild motor improvement reported	Small cohort; functional recovery modest and owner-reported	Prager et al. (2022)
Viral Vectors	Rat - Contusion at T10/11	Long-term ChABC expression; neuroprotection and improved motor function	Additional safety studies needed; risk of insertional mutagenesis; immune response to viral vector	Bartus et al. (2014)
	Rat - Contusion at C5	Upper limb motor improvement and axonal conduction	Off-target expression/possible limited control over duration (not evaluated)	James et al. (2015)
	Rat - Contusion at C5/C6	Recovered cortical plasticity; long term administration confers functional gains; determination of optimal therapeutic window	Lentiviral vectors - risk of mutagenesis; dox-inducible approach not translatable to the clinic	Burnside et al. (2018)
	Rat - Contusion at T10	Significant motor recovery, reduced glial scar; increased survival and migration of grafted cells	Lentiviral vector – risk of insertional mutagenesis; Transient PTEN silencing – limited long-term effect; Limited mechanistic validation; Dual genetic modification increases translational complexity	Lu et al. (2020)
	Mice	Cell-specific targeting of ChABC and long-term degradation of perineuronal nets	AAV diffusion contributes to non-specific spread; Immunogenicity of bacterial ChABC not addressed	Carstens et al. (2021)
Cell-based delivery	Dog - Compression at L4	Significant functional recovery	Additional anti-inflammatory strategy needed	Lee et al. (2015)
	Rat - Compression at C3	Significant motor improvement, superior functional recovery	Requires characterization of ChABC expression at intermediate time-points; neuroprotective effects were not explored	Prager et al. (2021)
	Dog - Chronic SCI (naturally occurring)	Safe and feasible; mild functional improvement reported by owners	Modest clinical outcomes; no objective gait measurement	Prager et al. (2022)

**TABLE 3:** Advantages and disadvantages of ChABC delivery methods.

Delivery strategy	Advantages	Disadvantages
Direct Administration of ChABC (intraparenchymal)	- Simple and direct method - Immediate dose control	- Rapid enzyme degradation - Requires repeated administration due to short half-life of ChABC - Potential adverse effects due to tissue damage associated with repeated administrations
Hydrogels and Biomaterials	- Sustained and localized release - Biocompatibility and protection against enzyme inactivation - Versatility for combined therapies: can be co-loaded with cells or drugs	- Requires surgical implantation - Difficult to precisely control release rate - Immune reactivity depending on composition
Nanoparticles	- Increased ChABC stability - Controlled release with specific triggers (pH, temperature) - Potential for non-invasive delivery	- Complex formulation process - Risk of off-target accumulation - Possible immunogenicity
mRNA-based delivery	- Transient, tunable expression - No genomic integration - Compatible with non-viral carriers	- Requires efficient delivery vehicle - Short half-life of mRNA - Potential innate immune activation
Viral Vectors (AAV, lentivirus)	- Sustained enzyme expression and release - Reduces need for repeated injections - Effective for large/charged molecules, where diffusion will be restricted due to their large molecular size	- Potential immunogenicity - Limited dose control - Potential long-term adverse effects/risk of insertional mutagenesis (for LV)
Cell therapy	- ChABC remodels ECM to favor cell migration - Cells can release trophic factors - Dual regenerative action	- Risk of immune rejection - Variable cell survival -Tumorigenic potential (source-dependent)

## 5 Recent advances and new technologies

A strategy developed by Lee and collaborators (2010) focused on stabilizing ChABC using sugar trehalose to prolong its activity and enhance thermal resistance. In their study, the modified enzyme remained active for 4 weeks at 37°C *in vitro* and retained its capacity to digest proteoglycans *in vivo* for 2 weeks following injury in a rat model of SCI (Lee et al., 2010). In another study, Hettiaratchi and collaborators (2020) adopted a different approach by computationally reengineering ChABC. They added 37, 55, and 92 amino acids using a hybrid consensus protein design, which relies on the most frequent amino acids in nature to increase protein stability over non-conserved residues (Porebski and Buckle, 2016). Their results showed that all three mutants were more stable than wild-type ChABC. Notably, the 37-residue mutant displayed the highest enzymatic activity, remaining active for 4.4 days and up to 7 days when combined with a hydrogel modified with SH3-binding peptides (Hettiaratchi et al., 2020). This study illustrates the growing importance of computational tools in addressing key challenges in protein stability and therapeutic efficacy.

Another promising technology in gene therapy is the use of messenger RNA (mRNA) delivery systems to promote *in vivo* production of therapeutically relevant proteins that are otherwise difficult to deliver. Khalil et al. (2022) demonstrated the use of non-viral mRNA-based delivery to express exogenous ChABC in a rat SCI model. ChABC synthesized from these mRNAs reduced proteoglycan deposition and improved motor function 7 weeks after injury, particularly when the microparticles were injected directly into the glial scar. This non-viral approach holds promise for circumventing immune responses associated with viral vectors while improving enzyme stability at the site of injury (Khalil et al., 2022). In another study, Hu et al. (2023) evaluated a combinatory strategy involving ChABC and knockdown of RhoA, a protein known to inhibit axonal growth following SCI. Using morpholino antisense oligonucleotides to silence RhoA expression in a lamprey SCI model, the researchers observed that combining RhoA knockdown with ChABC yielded the best neuroprotective effects. This combination significantly enhanced reticulospinal neuron survival, long-term axonal regeneration, inhibition of apoptotic signaling, and increased phosphorylation of pAkt-308. Although these results are promising, further electrophysiological studies are needed to confirm functional recovery (Hu et al., 2023).

Nanotechnology-based strategies have also been explored for the delivery of ChABC. Superparamagnetic iron oxide nanoparticles (SPIONs) can be functionalized with genetic material and directed by magnetic fields—a technique known as magnetofection—which enhances cellular uptake and spatial targeting (Zhao et al., 2014). SPIONs offer advantages such as protection of genetic material from nuclease degradation and, depending on their surface chemistry, relatively low immunogenicity. Although some SPION formulations are approved by the FDA for clinical applications like MRI contrast, their use in gene delivery remains experimental. In a study by Gao et al. (2020), SPIONs were coated with polyethyleneimine (PEI), a cationic polymer that facilitates DNA condensation and promotes endosomal escape, and used as a gene vector for ChABC. The authors combined this system with Schwann cells (SCs), which are known to secrete bioactive molecules that support axonal growth and provide permissive substrates for regeneration in spinal cord injury (SCI). SCs were magnetofected with PEI-SPIONs carrying the ChABC gene, and under a directional magnetic field, there was an 11.6-fold increase in SC localization to astrocyte-rich regions compared to controls. Furthermore, ChABC overexpression led to effective degradation of inhibitory proteoglycans, enhancing SC migration across the glial scar and promoting axonal regeneration following SCI.

In addition to nanoparticles, nanoclay biomaterials have also gained attention for their ability to modulate neuronal cell responses (Persano et al., 2021). Wu et al. (2024a) demonstrated that a ChABC-loaded nanoclay hydrogel could be delivered non-invasively to the injury site. Animals treated with this hydrogel showed decreased expression of inflammatory markers such as iNOS, GFAP and CS-56, as well as reduced scar formation. Furthermore, the treatment enhanced the survival of NF200+ neurons and promoted regeneration of Tuj-1+ neurons, leading to functional improvement (Wu et al., 2024b).

The use of low level laser (LLL) treatment for neurological disorders has also increased during the last years, because of the advantage of its use, as it is a non-invasive method with anti-inflammatory properties that are enhanced when combined with other therapies (Bertolini et al., 2011). So, in a work published by Janzadeh et al. (2017) they combined LLL with ChABC for the treatment of SCI. In this study, LLL + ChABC treatment was able to reduce glycogen synthase kinase-3β levels, which are known to be associated with the Wallerian degeneration and demyelination. Additionally, this group showed an increase in axonal outgrowth and remyelination, as well as an increase in motor function recovery in the rats, which was not observed when both treatments were used alone, as the LLL group still presented a deposition of fibrotic scar and the ChABC did not demonstrate an effect in the cytotoxic edema (Janzadeh et al., 2017). In another study, evaluating the effects of the combination between ChABC and hyperbaric oxygen therapy for SCI studied by Liu et al. (2018), they observed similar effects associated with glycogen synthase kinase-3β levels. The authors demonstrated that the combined treatment reduced the expression of this protein kinase and promoted myelination and functional recovery in rats after SCI (Liu et al., 2018).

Another promising method is the use of stem cells genetically modified for the delivery of ChABC, which is usually combined with other methods. Stem cells can be genetically engineered to deliver this enzyme in the system, with promising effects. Canine olfactory ensheathing cells modified to produce ChABC were able to digest chondroitin sulphate proteoglycans in a mouse model of spinal cord injury, leading to axonal regeneration (Carwardine et al., 2016). In another study, the combination of ChABC delivery with human induced pluripotent stem cell-derived neuroepithelial stem cells (NESCs) distributed in a hyaluronan and methylcellulose hydrogel was able to reduce cavity formation and an increase in the presence of neurons instead of oligodendrocytes and astrocytes was observed (Führmann et al., 2018). Thus, it seems that the combination between engineered stem cells to deliver ChABC and scaffolds or hydrogels seems to be the best method for ChABC delivery, as this could reduce the immunological effects that are present when viral vectors are used as a method of delivery and allow a long-term delivery of the enzyme in the injured area, promoting a better tissue recovery.

Therefore, these studies demonstrate the importance of the combination of treatments for the delivery of ChABC, as the enzyme used alone does not show promising effects, a fact that is not seen when combined with other therapies. These new combinations are able to induce inflammatory and apoptosis reduction, as well as an increase in axonal growth and functional recovery with the advantage of being methodologies that focus on reducing the invasiveness of the ChABC injections, a fact that is well received for clinical studies.

## 6 Future considerations and clinical applications

New potential therapies focusing on the ChABC delivery have emerged in recent years for the treatment of neurological disorders as outlined in this review. These studies, developed to optimize enzyme delivery, prevent degradation, and enhance regenerative potential, have significantly expanded our understanding of ChABCs duration of action and therapeutic efficacy when combined with other treatments. For instance, Mondello et al. (2015) demonstrated that cats treated with ChABC for 4 weeks showed an increased number of rubrospinal tract neurons with axons extending below the injury site. Similarly, Alilain and colleagues (2011) reported that ChABC administration at a chronic stage led to functional improvements in the respiratory motor system after cervical contusion. These findings highlight that ChABC’s therapeutic effects are substantially improved when used in conjunction with biomaterials, stem cells, or rehabilitation protocols, suggesting that the enzyme could play an essential role in future SCI therapies (Mondello et al., 2015; Alilain et al., 2011).

The molecular mechanisms by which ChABC promotes neuroplasticity and regeneration are still poorly described in the literature. The prolonged administration of ChABC contributed to the progressive motor function recovery in animals, as demonstrated by Burnside et al. (2018) as in their study, after 2–5 weeks, animals showed improved axonal sensory conduction and path performance. After 8 weeks, reaching and grasping abilities were observed, such as the rats ability to hold sugar pellets. Concurrently, these animals also exhibited increased vGlut1+ innervation density in the spinal cord rostral and caudal to the injury. The vGlut1+ neuronal population in the spinal cord consists of proprioceptive afferents that synapse onto motor neurons, corticospinal tract terminals, and primary afferent fibers. This vGlut1+ innervation may be associated with mechanisms underlying neuroplasticity. Future research should investigate the association between ChABC and neuroplasticity and tissue restoration mechanisms in greater detail. These insights may help advance the field and open up new therapeutic possibilities.

Regarding clinical translation, one major concern is the immune response to ChABC, as it is derived from *Proteus vulgaris*. The repeated administration of a foreign bacterial protein can trigger both innate and adaptive immune responses, leading to rapid neutralization of the enzyme or adverse local inflammation. However, the delivery strategies mentioned here not only enhance enzyme stabilization but also reduce ChABC’s immunogenic profile as various materials used in the construction of nanoparticles and hydrogels are biodegradable and exhibit low immunogenicity. Another example concerns gene therapies, which provide the gene to produce the enzyme locally and sustainably. It is also possible, through molecular engineering modifications, to carry out strategies such as enzymatic PEGylation, removal of immunogenic epitopes and other immunosuppressive regimens (Carstens et al., 2021).

Pharmacokinetic limitations, considering the short half-life of ChABC, significantly hinder the viability of ChABC as a stand-alone injectable therapy in clinical settings, especially for chronic SCI. Thus, the use of delivery systems is a good alternative to increase enzymatic stability and promote sustained delivery. Different nanoparticle and hydrogel materials can help to maintain the system preserved and more efficient, such as the previously mentioned self-assembling peptide-based hydrogels and PLGA nanoparticles, ideal for chronic models (Raspa et al., 2021; Azizi et al., 2020). In particular, viral vectors have been shown to preserve ChABC activity for approximately weeks, with transduced cells synthesizing the enzyme continuously, which would solve the clinical need for multiple administrations (Bartus et al., 2014; James et al., 2015).

However, the effects of ChABC treatment in humans still remain to be fully explored, as there are no clinical trials evaluating a treatment with this enzyme. While advancements have been made in ChABC delivery and its impact on central nervous system regeneration—particularly in SCI—there is a noticeable gap in the literature regarding innovation and replication of results. For example, low-level laser therapy seems to be a promising therapy when combined with ChABC demonstrating interesting results (Janzadeh et al., 2017), however, we observed only one published article using this combination in a non-SCI model (Sarveazad et al., 2021).

Although ChABC has not been tested in human clinical trials, a notable preclinical study by Rosenzweig et al. (2019) investigated its effects in rhesus monkeys following a C7 spinal cord hemisection. In this study they observed that 4 weeks after the injury, multiple injections of ChABC were able to promote hand function improvement and in corticospinal axon growth (Rosenzweig et al., 2019). However, as said before, the use of multiple injections is not preferred when taking into consideration the translation of this therapy for the clinic, as it can cause tissue damage. Additionally, even if a continuous delivery of ChABC does not seem to be associated with any adverse effects after 8 weeks in rats (Pakulska et al., 2017), the levels of proteoglycans demand some time to return to normal levels (Gordon et al., 2010), a fact that will need to be monitored in animals over a long period of time in order to observe possible adverse effects.

In summary, ChABC represents a promising therapeutic strategy for spinal cord injury due to its capacity to degrade proteoglycans, thereby facilitating neural regeneration and functional recovery. Combined therapies involving ChABC—whether with biomaterials, stem cells, or rehabilitation techniques—have shown greater efficacy than the enzyme alone. However, the current lack of robust and replicable preclinical studies hinders the transition of ChABC-based approaches to the clinic. Therefore, future research should prioritize translational studies that bridge the gap between experimental success and clinical implementation, ultimately aiming to improve outcomes for patients with chronic SCI.

## Abbreviations

SCI, spinal cord injury; CNS, central nervous system; CSPGs, chondroitin sulfate proteoglycans; ChABC, Chondroitinase ABC; BSCB, blood-spinal cord barrier; ECM, extracellular matrix; GFAP, glial fibrillary acidic protein; GSK-3β, glycogen synthase kinase-3β; PLGA, poly (lactic-co-glycolic acid); BBB, Basso Beattie and Bresnahan; SAPs, self-assembling peptide hydrogels; Gal3, galectin-3; HA, hyaluronan; mOEC, mucosal olfactory ensheathing cell; LVs, lentiviral vectors; AAVs, adeno-associated viruses; NSCs, neural stem cells; iPSC, induced pluripotent stem cells; oNPCs, oligodendrogenic neural progenitor cells; SCs, schwann cells; MSCs, Mesenchymal stem/stromal cells; ADSCs, adipose-derived mesenchymal stem cells; mRNA, messenger RNA; SPIONs, superparamagnetic ferric oxide nanoparticles; PEI, polyethyleneimine; LLL, low level laser; NESCs; stem cell-derived neuroepithelial stem cells.
